# Association of physical activity and dietary inflammatory index with overweight/obesity in US adults: NHANES 2007–2018

**DOI:** 10.1265/ehpm.23-00016

**Published:** 2023-06-28

**Authors:** Jikang Shi, Zhuoshuai Liang, Xin Zhang, Shuping Ren, Yi Cheng, Yawen Liu, Ming Zhang

**Affiliations:** 1 Department of Clinical Nutrition, Peking University Shenzhen Hospital, Shenzhen 518036, China; 2 Department of Epidemiology and Biostatistics, School of Public Health of Jilin University, Changchun 130021, China; 3 The Cardiovascular Center, The First Hospital of Jilin University, Changchun 130021, Jilin, China

**Keywords:** Overweight/obesity, Physical activity, Dietary inflammatory index, NHANES

## Abstract

**Background:**

Overweight and obesity lead to a range of noncommunicable diseases (NCDs), such as type 2 diabetes, cardiovascular disease, and stroke. Physical activity (PA) is an important lifestyle behavior for controlling body weight. Dietary inflammatory index (DII), which is associated with systemic inflammatory markers, is used to evaluate the potential of dietary inflammation. This is the first study to investigate the independent and joint associations of PA and DII with the risk of overweight/obesity among US adults.

**Methods:**

Participants and data were obtained from the National Health and Nutrition Examination Survey (NHANES) from 2007–2018, which is designed to examine the health and nutritional status of the non-institutionalized US population by a complex, multi-stage, probability sampling design.

**Results:**

A total of 10723 US adults were selected. Physically active participants had lower overweight/obesity risk (total-time PA: OR = 0.756, 95% CI: 0.669–0.855; leisure-time PA: OR = 0.723, 95% CI: 0.643–0.813; and walk/bicycle-time PA: OR = 0.748, 95% CI: 0.639–0.875); however, those with work-time PA showed no significant association between PA and overweight/obesity. Compared with participants in the lowest DII group (Q1), those in the other three groups had high risks of overweight/obesity (Q2: OR = 1.218, 95% CI: 1.054–1.409; Q3: OR = 1.452, 95% CI: 1.245–1.693; Q4: OR = 1.763, 95% CI: 1.495–2.079). In joint analyses, PA was not eligible for reducing risks of weight/obesity if far more pro-inflammatory diet (Q4 of DII = 2.949–5.502) was taken in (total-time PA: OR = 1.725, 95% CI: 1.420–2.097; leisure-time PA: OR = 1.627, 95% CI: 1.258–2.105; walk/bicycle-time PA: OR = 1.583, 95% CI: 1.074–2.332; and work-time PA: OR = 1.919, 95% CI: 1.493–2.467).

**Conclusions:**

More leisure-time PA and walk/bicycle-time PA are associated with lower risk of overweight/obesity, and higher DII is associated with higher risk of overweight/obesity. In addition, higher DII impacts overweight/obesity substantially: once the DII score reached Q4, there is still risks of overweight/obesity even if PA is performed.

**Supplementary information:**

The online version contains supplementary material available at https://doi.org/10.1265/ehpm.23-00016.

## 1. Introduction

Overweight and obesity are defined as abnormal or excessive fat accumulation, leading to risks for health [1]. According to the global burden of disease, there were more than 4 million people dying each year owing to being overweight or obese in 2017 [2]. Moreover, there were more than 1.9 billion overweight adults and 650 million obese adults by 2019 [3] and the number of overweight or obese people may reach 3.3 billion people worldwide in 2030 [4]. Overweight and obesity give rise to a range of noncommunicable diseases (NCDs), such as type 2 diabetes, cardiovascular disease, hypertension and stroke, various forms of cancer, and mental health issues [5–7].

Physical activity (PA) is an important lifestyle behavior that controls weight, improves cardiorespiratory fitness, and reduces overall mortality [8–10]. PA is associated with obesity even though the exact etiology of obesity is unclear [11]. Lack of PA affords more accumulation of fat, especially visceral fat, promoting inflammation [12]. However, different types of PA produce different effects on health, such as leisure-time PA, walk-time PA, and work-time PA [13].

Dietary inflammatory index (DII), which is associated with systemic inflammatory markers, is used to evaluate the potential of dietary inflammation [14, 15]. DII is developed as a standardized scoring system to assess intakes of 45 pro-inflammatory and anti-inflammatory dietary components. A higher DII score represents a pro-inflammatory diet, whereas a lower DII score represents an anti-inflammatory diet [16]. In addition, DII is associated with inflammatory-related conditions, such as obesity, cardiovascular disease, cancers, and mortality [17–21].

Overweight and obesity are associated with development of long-term inflammation and low PA and poor diet also contribute to the occurrence of chronic inflammation [22, 23]. Regular PA and healthy diet are essential for keeping weight [24]. The effect of PA or DII on overweight/obesity has been investigated separately. However, the joint effect of PA and DII remains obscure. Unveiling the joint effect is an integral step of crystallizing the associations of PA and DII with the risk of overweight/obesity. In this paper, we investigated the independent and joint associations of PA and DII with the risk of overweight/obesity among adult population using the data from the National Health and Nutrition Examination Surveys (NHANES).

## 2. Materials and methods

### 2.1. Participants and data

Participants and data were obtained from the National Health and Nutrition Examination Survey (NHANES), a cross-sectional survey program conducted in the United States by the National Center for Health Statistics (NCHS) at the US Centers for Disease Control and Prevention. NHANES is designed to examine the health and nutritional status of the non-institutionalized US population using a complex, multi-stage, probability sampling design. Participants were interviewed in their homes and then subsequently examined in a mobile examination center (MEC). Further information on NHANES methodology and data collection is available on the NHANES website [25].

The participants selected in this study were the adults aged 18 years or older from 2007–2018 cycles of NHANES. In addition, participants met following criteria were excluded: (1) body mass index (BMI) < 18.5 kg/m^2^; (2) with major chronic diseases including hypertension, diabetes, coronary heart disease, stroke, and cancer; (3) without complete data information.

### 2.2. Exposure and outcome variables

Overweight and obesity in adults were classified by BMI and the BMI was defined as weight in kilograms divided by the square of height in meters (kg/m^2^). Body weight status in this study was classified as follows: healthy weight (BMI = 18.5–24.9), and overweight and obesity (BMI ⩾ 25) [1].

Physical activity (PA) was divided into four types: total-time PA, leisure-time PA (any physical activity for the purpose of recreation and/or fitness), walk/bicycle-time PA (transport to/from work or school), and work-time PA (work-related PA). According to the physical activity guideline from the American College of Sports Medicine (ACSM), active PA was defined as 150 min of moderate PA or 75 min of vigorous PA; otherwise, inactive PA was defined [26, 27].

Dietary inflammatory index (DII) was calculated using the 24-h dietary recall interviews (24HR) [28]. This index was developed on the basis of 45 food parameters. Inflammatory biomarkers (IL-1b, IL-4, IL-6, IL-10, TNF-a and C-reactive protein) are used to establish the DII. If a kind of food increased IL-1β, IL-6, TNF-α or CRP, or decreased IL-4 or IL-10, ‘+1’ was assigned for the food parameter on inflammation; if a kind of food significantly decreased IL-1β, IL-6, TNF or CRP, or increased IL-4 or IL-10, ‘-1’ was assigned; and if the food parameter did not produce any significant change in the inflammatory marker, ‘0’ was assigned. DII is the sum of food parameters on inflammation [16]. Moreover, DII score was categorized into quartiles: Q1 indicates anti-inflammatory diet; additionally, Q2, Q3, and Q4 indicate pro-inflammatory diet with increasing intensity. The anti-inflammatory group (Q1) was used as the reference category.

### 2.3. Other variables

General characteristics of participants included sex (male or female), race and ethnicity (Hispanic, non-Hispanic White, non-Hispanic Black, and other race), family poverty income ratio (poor: <1.3, median: 1.3–3.49, and rich: ⩾ 3.5), education (less than high school graduate, high school graduate or general educational development [GED], and some college or above), marital status (married, never married, widowed, divorced, separated, and living with partner), smoking (never, former, and now), and drinking (never, former, mild, moderate, and heavy).

### 2.4. Statistical analysis

Statistical analyses were performed under the complex sampling weight of NHANES according to the CDC guidelines [29]. For weighted characteristics description, continuous variables were presented as mean ± standard error (SE) and categorical variables were presented as percentages. Generalized linear model was performed to analyze odds ratios (OR) and 95% confidence interval (CI) for overweight/obesity and the joint association of PA and DII with overweight/obese on the basis of the Model 1 (unadjusted), Model 2 (age and sex were adjusted), and Model 3 (sex, age, race/ethnicity, family poverty income ratio, education, marital status, smoking, and drinking were adjusted). In addition, we performed stratified analyses to evaluate the association of PA and DII with overweight/obese according to sex. All analyses were performed using the statistical software packages R (The R Foundation; version 4.2.1) and EmpowerStats (www.empowerstats.net, X&Y solutions, Inc., Boston, Massachusetts), and *P*-values < 0.05 was considered statistically significant.

## 3. Results

### 3.1. General characteristics of participants

A total of 10723 US adults (3827 participants with normal weight and 6896 participants with overweight/obesity) were selected in this study, with mean age of the participants was 40.8 years. There existed different ratios among participants with PA (total-time PA: 73.0%, leisure-time PA: 45.9%, walk/bicycle-time PA: 14.3%, and work-time PA: 40.9%). DII score ranged from a maximum anti-inflammatory value of −4.634 to a maximum pro-inflammatory value of +5.502. DII score was categorized into quartiles (Q1, Q2, Q3, and Q4): Q1 (−4.634 to 0.061), Q2 (0.062–1.625), Q3 (1.626–2.948), and Q4 (2.949–5.502). The corresponding numbers of participants affiliating the quartiles are listed in Table 1. Moreover, the general characteristics of participants by sex and weight status are presented in Supplementary Table S1.

**Table 1:** General characteristics of participants by weight status (Mean ± SE/N(weighted%))

**Characteristics**	**Total**	**Normal weight**	**Overweight/obese**
**Overall**	10723	3827	6896
**Age**	40.8 ± 14.9 (40.8)	39.3 ± 15.3 (39.5)	41.6 ± 14.6 (41.6)
**sex**			
Female	5640 (52.8)	2095 (58.2)	3545 (49.7)
Male	5083 (47.2)	1732 (41.8)	3351 (50.3)
**Race/ethnicity**			
Hispanic group	2817 (14.7)	711 (10.2)	2106 (17.3)
Non-Hispanic White	4723 (69.1)	1801 (72.3)	2922 (67.3)
Non-Hispanic Black	1861 (9.0)	575 (7.5)	1286 (9.8)
Other Race	1322 (7.2)	740 (10.0)	582 (5.6)
**Family poverty income ratio**			
<1.30	3146 (19.5)	1066 (19.1)	2080 (19.7)
1.30–3.49	3900 (33.7)	1316 (30.5)	2584 (35.5)
≥3.50	3677 (46.8)	1445 (50.4)	2232 (44.8)
**Education**			
Less than high school graduate	1896 (11.6)	544 (9.5)	1352 (12.8)
High school graduate or GED	2271 (20.7)	736 (18.0)	1535 (22.3)
Some college or above	6556 (67.7)	2547 (72.5)	4009 (64.9)
**Marital status**			
Married	5379 (54.3)	1830 (51.2)	3549 (56.1)
Never married	2680 (23.6)	1162 (28.7)	1518 (20.7)
Widowed	276 (1.9)	78 (1.5)	198 (2.2)
Divorced	947 (8.7)	300 (7.9)	647 (9.2)
Separated	312 (2.0)	99 (2.0)	213 (2.1)
Living with partner	1125 (9.4)	356 (8.8)	769 (9.7)
**Smoking**			
Never	6523 (60.8)	2388 (62.5)	4135 (59.8)
Former	1994 (20.3)	596 (17.6)	1398 (21.9)
Now	2206 (18.9)	843 (19.9)	1363 (18.3)
**Drinking**			
Never	1336 (9.6)	490 (9.5)	846 (9.7)
Former	1143 (9.2)	347 (7.3)	796 (10.3)
Mild	3660 (36.9)	1374 (38.3)	2286 (36.1)
Moderate	2002 (20.2)	739 (22.2)	1263 (19.0)
Heavy	2582 (24.1)	877 (22.7)	1705 (24.8)
**Total-Time PA**			
Inactive	3275 (27.0)	1059 (23.3)	2216 (29.1)
Active	7448 (73.0)	2768 (76.7)	4680 (70.9)
**Leisure-Time PA**			
Inactive	6247 (54.1)	2037 (47.9)	4210 (57.7)
Active	4476 (45.9)	1790 (52.1)	2686 (42.3)
**Walk/Bicycle-Time PA**			
Inactive	9018 (85.7)	3104 (83.2)	5914 (87.1)
Active	1705 (14.3)	723 (16.8)	982 (12.9)
**Work-Time PA**			
Inactive	6552 (59.1)	2425 (61.0)	4127 (58.0)
Active	4171 (40.9)	1402 (39.0)	2769 (42.0)
**Dietary inflammatory index (quartile)**			
Q1	2899 (29.1)	1170 (33.0)	1729 (26.9)
Q2	2759 (25.7)	1004 (26.3)	1755 (25.4)
Q3	2575 (23.8)	871 (22.5)	1704 (24.5)
Q4	2490 (21.4)	782 (18.2)	1708 (23.2)

DII quartile ranges: Quartile 1 = −4.634 to 0.061, Quartile 2 = 0.062–1.625, Quartile 3 = 1.626–2.948, Quartile 4 = 2.949–5.502.

### 3.2. Association of PA with overweight/obesity

**Fig. 1:**
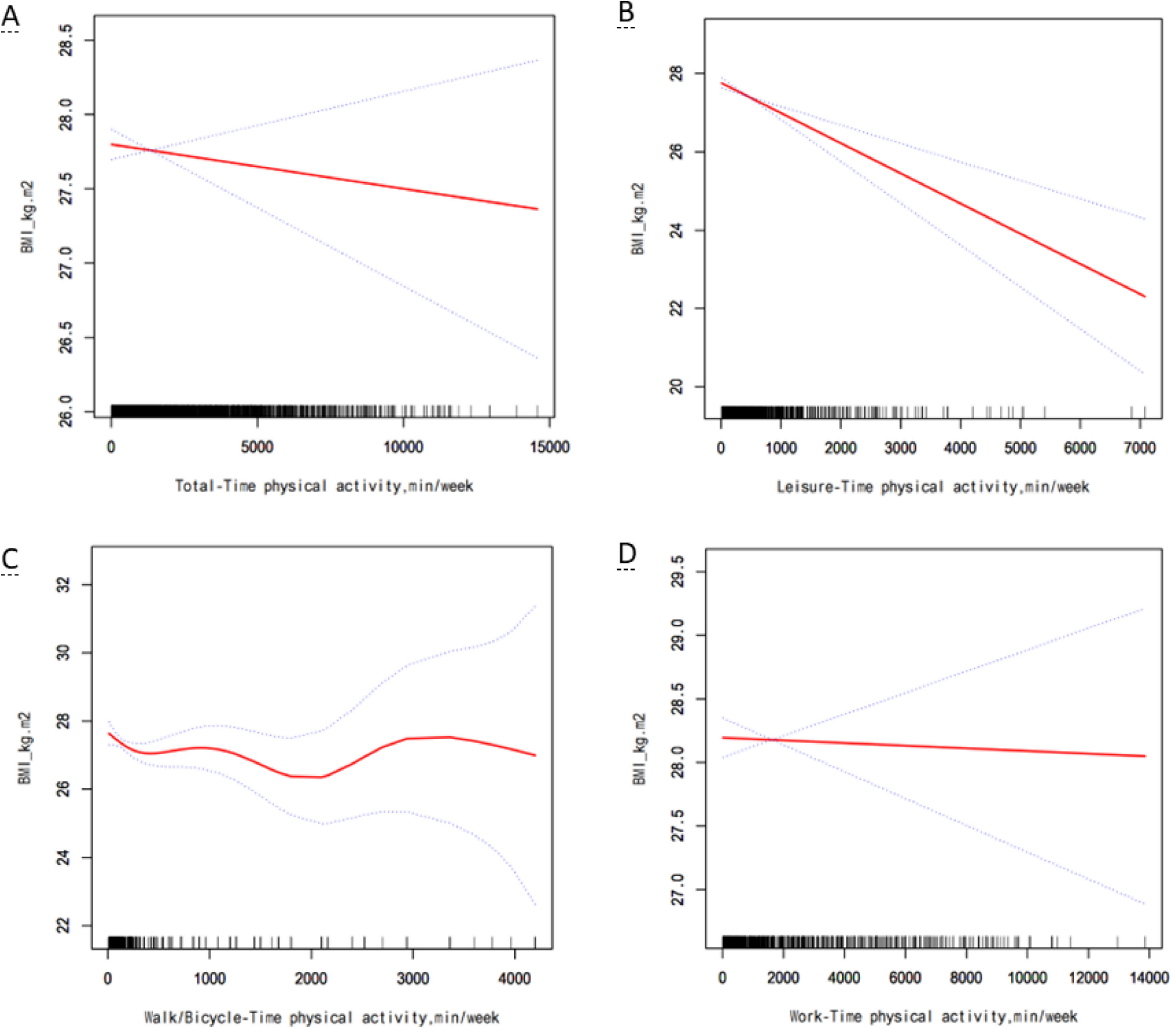
Multivariate adjusted smoothing spline plots of PA time and BMI. This model was adjusted for sex, age, race/ethnicity, family poverty income ratio, education, marital status, smoking, and drinking. (A) total-time PA; (B) leisure-time PA; (C) walk/bicycle-time PA; (D) work-time PA.

Physically active participants had lower risks of overweight/obesity than inactive participants (total-time PA: OR = 0.739, 95% CI: 0.657–0.831; leisure-time PA: OR = 0.674, 95% CI: 0.603–0.754; and walk/bicycle-time PA: OR = 0.731, 95% CI: 0.633–0.845). After adjusting variables (sex, age, race/ethnicity, family poverty income ratio, education, marital status, smoking, and drinking), physically active participants were still of lower risks of overweight/obesity (total-time PA: OR = 0.756, 95% CI: 0.669–0.855; leisure-time PA: OR = 0.723, 95% CI: 0.643–0.813; and walk/bicycle-time PA: OR = 0.748, 95% CI: 0.639–0.875). However, there was no significant association between work-time PA and overweight/obesity (OR = 1.063, 95% CI: 0.934–1.210) (Table 2). Association between PA and overweight/obesity in total participants were consistent with that in female participants but not in male participants (Supplementary Table S2). In addition, there were negatively linear dose-response nexus between leisure-time PA and BMI both in total participants and in participants stratified by sex significantly (*P* < 0.01) (Fig. 1, Supplementary Figure F1, and Supplementary Figure F2).

**Table 2:** Association of physical activity with overweight/obese

**Physical activity (PA)**	**Model 1**	**Model 2**	**Model 3**

	**OR (95%CI)**	**OR (95%CI)**	**OR (95%CI)**
**Total-Time PA**			
Inactive	1.000 (reference)	1.000 (reference)	1.000 (reference)
Active	**0.739 (0.657, 0.831)**	**0.715 (0.635, 0.806)**	**0.756 (0.669, 0.855)**
**Leisure-Time PA**			
Inactive	1.000 (reference)	1.000 (reference)	1.000 (reference)
Active	**0.674 (0.603, 0.754)**	**0.673 (0.602, 0.753)**	**0.723 (0.643, 0.813)**
**Walk/Bicycle-Time PA**			
Inactive	1.000 (reference)	1.000 (reference)	1.000 (reference)
Active	**0.731 (0.633, 0.845)**	**0.729 (0.628, 0.847)**	**0.748 (0.639, 0.875)**
**Work-Time PA**			
Inactive	1.000 (reference)	1.000 (reference)	1.000 (reference)
Active	1.136 (0.999, 1.291)	1.106 (0.973, 1.258)	1.063 (0.934, 1.210)

Model 1: no covariates were adjusted.

Model 2: adjusted for sex and age.

Model 3: adjusted for sex, age, race/ethnicity, family poverty income ratio, education, marital status, smoking, and drinking.

### 3.3. Association of DII with overweight/obesity

**Fig. 2:**
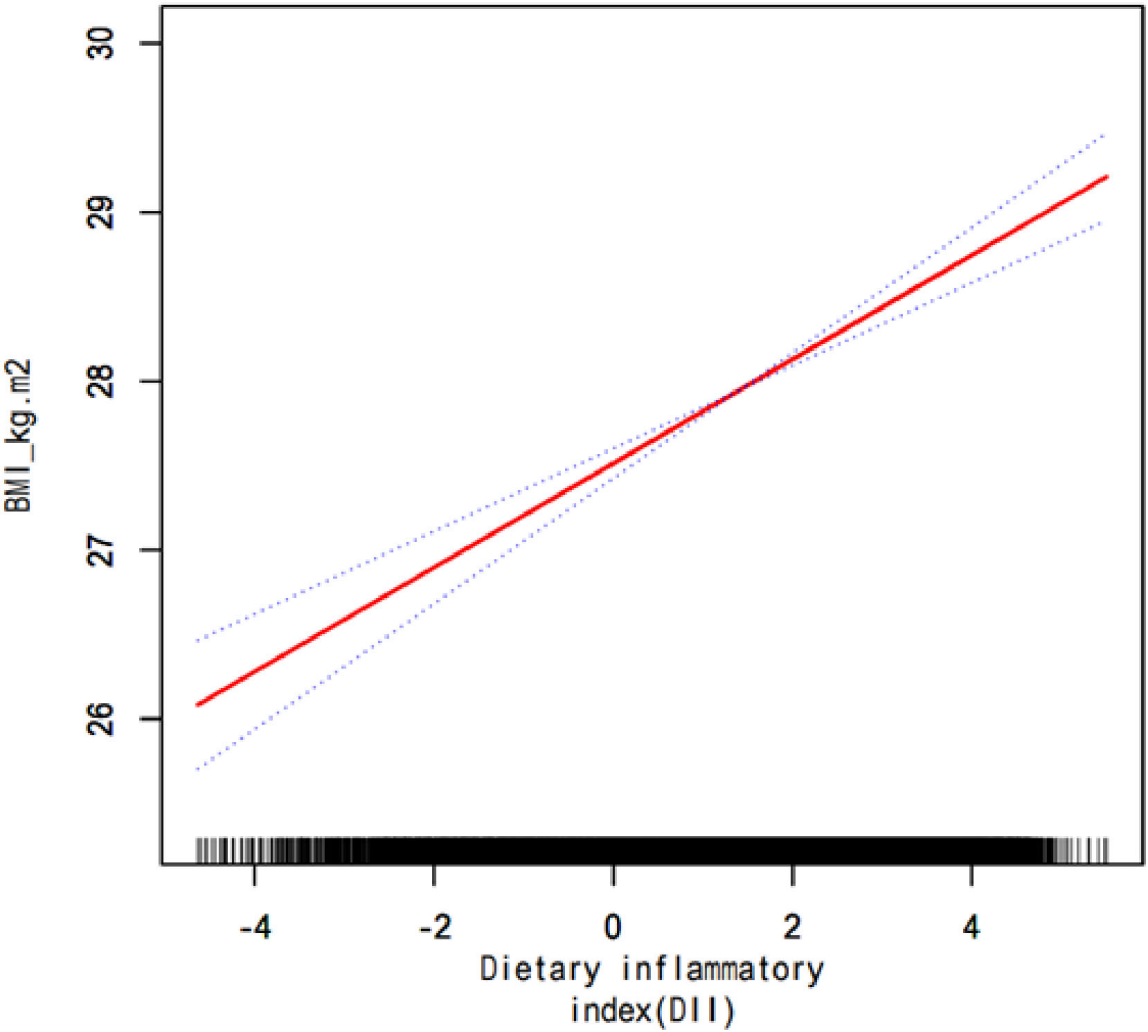
Multivariate adjusted smoothing spline plots of DII and BMI. This model was adjusted for sex, age, race/ethnicity, family poverty income ratio, education, marital status, smoking, and drinking.

Compared with participants in the lowest DII group (Q1), those in the other three groups had high risks of overweight/obesity (Q2: OR = 1.187, 95% CI: 1.033–1.364; Q3: OR = 1.333, 95% CI: 1.156–1.539; and Q4: OR = 1.562, 95% CI: 1.326–1.840). After adjusting for all of the variables (sex, age, race/ethnicity, family poverty income ratio, education, marital status, smoking, and drinking), high DII still conferred independent risks for overweight/obesity (Q2: OR = 1.218, 95% CI: 1.054–1.409; Q3: OR = 1.452, 95% CI: 1.245–1.693; and Q4: OR = 1.763, 95% CI: 1.495–2.079) (Table 3). Moreover, DII was positively associated with the BMI (*P* < 0.01) (Fig. 2). We further investigated the association between the DII and the risk of overweight/obesity among participants stratified by sex, finding that, compared with female participants in the lowest DII group, those in the other three groups (Q2, Q3, and Q4) had high risks of overweight/obesity; of note, compared with male participants in the lowest DII group, those from the Q3 and Q4 had high risks of overweight/obesity (Supplementary Table S3). Additionally, significant positive dose-response nexus between DII and BMI were found both in female and in male participants (*P* < 0.01) (Supplementary Figure F3 and Supplementary Figure F4).

**Table 3:** Association of dietary inflammatory index with overweight/obese

**Dietary inflammatory** **index (quartile)**	**Model 1**	**Model 2**	**Model 3**

	**OR (95%CI)**	**OR (95%CI)**	**OR (95%CI)**
**Q1**	1.000 (reference)	1.000 (reference)	1.000 (reference)
**Q2**	**1.187 (1.033, 1.364)**	**1.265 (1.101, 1.452)**	**1.218 (1.054, 1.409)**
**Q3**	**1.333 (1.156, 1.539)**	**1.484 (1.284, 1.715)**	**1.452 (1.245, 1.693)**
**Q4**	**1.562 (1.326, 1.840)**	**1.821 (1.538, 2.156)**	**1.763 (1.495, 2.079)**

Model 1: no covariates were adjusted.

Model 2: adjusted for sex and age.

Model 3: adjusted for sex, age, race/ethnicity, family poverty income ratio, education, marital status, smoking, and drinking.

DII quartile ranges: Quartile 1 = −4.634 to 0.061, Quartile 2 = 0.062–1.625, Quartile 3 = 1.626–2.948, Quartile 4 = 2.949–5.502.

### 3.4. Joint association of PA and DII with overweight/obesity

For physically inactive group, compared with participants in the lowest DII group (Q1), those in the other three groups (Q2, Q3, and Q4) had high risks of overweight/obesity (for leisure-time PA, Q2: OR = 1.239, 95% CI: 1.044–1.471; Q3: OR = 1.425, 95% CI: 1.144–1.777; Q4: OR = 1.882, 95% CI: 1.569–2.259) (for walk/bicycle-time PA, Q2: OR = 1.238, 95% CI: 1.062–1.443; Q3: OR = 1.490, 95% CI: 1.248–1.779; Q4: OR = 1.813, 95% CI: 1.508–2.180) (for work-time PA, Q2: OR = 1.295, 95% CI: 1.062–1.578; Q3: OR = 1.533, 95% CI: 1.277–1.839; Q4: OR = 1.665, 95% CI: 1.363–2.034) after adjusting for all of the variables.

However, for physically active group, compared with participants in Q1 group, those in Q2 group had not risks for overweight/obesity, those in the groups (Q3 and Q4) had high risks of overweight/obesity (for leisure-time PA, Q3: OR = 1.505, 95% CI: 1.178–1.923; Q4: OR = 1.627, 95% CI: 1.258–2.105) (work-time PA, Q3: OR = 1.335, 95% CI: 1.046–1.704; Q4: OR = 1.919, 95% CI: 1.493–2.467). For walk/bicycle-time PA, only participants in Q4 group had higher risks of overweight/obesity than those in Q1 group (Q4: OR = 1.583, 95% CI: 1.074–2.332) (Table 4). Surprisingly, for leisure-time PA and work-time PA, more PA linked to greater risks of overweight/obesity than less PA among women in Q4 group (Supplementary Table S4).

**Table 4:** Joint association of physical activity and dietary inflammatory index with overweight/obese

**Physical activity (PA)**	**DII** **(quartile)**	**Model 1**	**Model 2**	**Model 3**

		**OR (95%CI)**	**OR (95%CI)**	**OR (95%CI)**
**Total-Time PA**				
Inactive	Q1	1.000 (reference)	1.000 (reference)	1.000 (reference)
	Q2	0.977 (0.780, 1.223)	0.998 (0.796, 1.253)	1.279 (0.997, 1.643)
	Q3	1.277 (0.982, 1.660)	**1.319 (1.025, 1.699)**	**1.726 (1.262, 2.361)**
	Q4	1.204 (0.900, 1.611)	1.271 (0.952, 1.695)	**1.817 (1.389, 2.377)**
Active	Q1	1.000 (reference)	1.000 (reference)	1.000 (reference)
	Q2	**1.214 (1.034, 1.425)**	**1.305 (1.114, 1.528)**	1.191 (1.004, 1.414)
	Q3	**1.268 (1.071, 1.501)**	**1.448 (1.216, 1.725)**	**1.357 (1.150, 1.600)**
	Q4	**1.624 (1.344, 1.961)**	**1.960 (1.609, 2.386)**	**1.725 (1.420, 2.097)**
**Leisure-Time PA**				
Inactive	Q1	1.000 (reference)	1.000 (reference)	1.000 (reference)
	Q2	1.207 (0.989, 1.472)	**1.261 (1.032, 1.542)**	**1.239 (1.044, 1.471)**
	Q3	**1.297 (1.062, 1.585)**	**1.397 (1.147, 1.701)**	**1.425 (1.144, 1.777)**
	Q4	**1.392 (1.114, 1.738)**	**1.538 (1.230, 1.923)**	**1.882 (1.569, 2.259)**
Active	Q1	1.000 (reference)	1.000 (reference)	1.000 (reference)
	Q2	1.072 (0.881, 1.306)	1.141 (0.938, 1.389)	1.197 (0.952, 1.506)
	Q3	1.206 (0.972, 1.495)	**1.373 (1.103, 1.709)**	**1.505 (1.178, 1.923)**
	Q4	**1.547 (1.246, 1.922)**	**1.899 (1.511, 2.387)**	**1.627 (1.258, 2.105)**
**Walk/Bicycle-Time PA**				
Inactive	Q1	1.000 (reference)	1.000 (reference)	1.000 (reference)
	Q2	1.115 (0.956, 1.300)	**1.189 (1.019, 1.388)**	**1.238 (1.062, 1.443)**
	Q3	**1.311 (1.112, 1.547)**	**1.464 (1.236, 1.735)**	**1.490 (1.248, 1.779)**
	Q4	**1.490 (1.238, 1.793)**	**1.736 (1.432, 2.105)**	**1.813 (1.508, 2.180)**
Active	Q1	1.000 (reference)	1.000 (reference)	1.000 (reference)
	Q2	**1.600 (1.119, 2.287)**	**1.680 (1.168, 2.418)**	1.149 (0.820, 1.612)
	Q3	1.388 (0.991, 1.947)	**1.532 (1.096, 2.141)**	1.335 (0.953, 1.869)
	Q4	**1.909 (1.404, 2.596)**	**2.288 (1.678, 3.121)**	**1.583 (1.074, 2.332)**
**Work-Time PA**				
Inactive	Q1	1.000 (reference)	1.000 (reference)	1.000 (reference)
	Q2	**1.189 (1.004, 1.408)**	**1.273 (1.076, 1.507)**	**1.295 (1.062, 1.578)**
	Q3	**1.494 (1.247, 1.789)**	**1.647 (1.371, 1.979)**	**1.533 (1.277, 1.839)**
	Q4	**1.576 (1.307, 1.901)**	**1.826 (1.511, 2.208)**	**1.665 (1.363, 2.034)**
Active	Q1	1.000 (reference)	1.000 (reference)	1.000 (reference)
	Q2	1.184 (0.942, 1.489)	1.251 (0.996, 1.572)	1.102 (0.887, 1.369)
	Q3	1.131 (0.882, 1.449)	1.271 (0.991, 1.633)	**1.335 (1.046, 1.704)**
	Q4	**1.543 (1.202, 1.980)**	**1.810 (1.403, 2.336)**	**1.919 (1.493, 2.467)**

Model 1: no covariates were adjusted.

Model 2: adjusted for sex and age.

Model 3: adjusted for sex, age, race/ethnicity, family poverty income ratio, education, marital status, smoking, and drinking.

DII quartile ranges: Quartile 1 = −4.634 to 0.061, Quartile 2 = 0.062–1.625, Quartile 3 = 1.626–2.948, Quartile 4 = 2.949–5.502.

## 4. Discussions

In this study, we identified that participants with more leisure-time PA or with more walk/bicycle-time PA had lower risks of overweight/obesity; however, higher DII was associated with higher risks of overweight/obesity. Moreover, the joint of higher DII and more PA still was associated with risks of overweight/obesity, indicating that higher DII (pro-inflammatory diet) impacts overweight/obesity substantially.

We found that leisure-time PA and walk/bicycle-time PA were associated with reduced risks of overweight/obesity, but there was no significant association between work-time PA and overweight/obesity. Schmitz et al. also found that change in PA is inversely associated with change in body weight [30]. Sarma et al. found that an active level of leisure-time PA reduced the probability of obesity [31]. Gordon-Larsen et al. demonstrated that walk/bicycle-time PA decreases obesity [32]. Unexpectedly, Monda et al. and Steeves et al. showed that increased work-time PA results in lower body weight [33, 34], whereas Larsson et al. found that work-time PA is positively associated with BMI [35]. Paradoxically, Gutiérrez-Fisac et al. and us found no association between work-time PA with risks of obesity [36]. Types of PA affect overweight/obesity to different extent. Leisure-time PA and walk/bicycle-time PA have protective effects on overweight/obesity, partially owing to their purpose of fitness or weight loss. In contrast, work-time PA affiliates a kind of forced exercise; thus, inconsistent results reflect different load intensity and attitudes towards this forced exercise in populations investigated in literatures. Excessive load intensity may have a negative effect.

Compared with the anti-inflammatory diet (Q1), the pro-inflammatory diets (Q2, Q3, and Q4) were associated with increased risks of overweight/obesity in this study. The highest DII is associated with the highest risk of overweight/obesity [37]. Moreover, both Ruiz-Canela et al. [18] and us find positive dose-response nexus between BMI and DII. Inflammatory cytokines may link proinflammatory diet to overweight/obesity. Pro-inflammatory foods, including high sugar foods, refined grains, red and processed meats, fried foods, soft drinks, sugar, and sweets can increase levels of inflammatory cytokines, such as IL-6, IL-1, TNF-α, and CRP [38, 39]. In contrast, anti-inflammatory foods, such as dairy products, white and lean meats, fish/shellfish, eggs, whole grains, nuts, fruits, and vegetables, decreases levels of the inflammatory cytokines [24]. Moreover, the inflammatory cytokines induce weight gain [40, 41], partially because the proinflammatory cytokines can stimulate appetite, thereby increasing energy intake and fat deposition [42].

Our joint analyses revealed PA was not eligible for reducing risks of weight/obesity if far more pro-inflammatory diet was taken in. Higher DII and more PA were still associated with risks of overweight/obesity, indicating that higher DII (pro-inflammatory diet) impacts overweight/obesity substantially. However, the joint of higher DII and more PA decreased risk of overweight/obesity than that of higher DII and less PA, indicating PA is necessary for preventing weight gain. Regular PA can decrease the inflammation through enhancing the production of anti-inflammation environment and reducing visceral fat which produces pro-inflammatory adipokines such as IL-6 and TNF-α [43]. Therefore, the joint of lower DII (anti-inflammatory diet) and more PA contribute to preventing overweight/obesity.

Moreover, we found that women were more sensitive to the changes in weight/obesity. PA reduced the risk of weight/obesity for both male and female, especially for female. Conversely, pro-inflammatory diet enhanced the risk of weight/obesity for both male and female: this diet became a risk factor for female when DII reached Q2; however, that became a risk factor for male when DII reached Q3. In addition, under the condition of co-existence of PA and pro-inflammatory diet, walk/bicycle PA was a protective factor for female, but leisure-time PA was a protective factor for male. These may reflect hormonal difference and interactions between genes and environment. Additionally, physical environment affects the effectiveness of physical activity [44]; moreover, the joint effect of physical activity and physical environment substantiates changes of health outcome [45]. There exist different skeletal muscle mass indices (SMI) between male and female: SMI of male is greater than that of female. Because female usually store much fat than male, female is liable to the effect of physical activity and pro-inflammatory diet [46]. Of note, PA was not a protective factor when DII reached Q4. Thus, pro-inflammatory diet impacts overweight/obesity substantially.

To our knowledge, this is the first study to evaluate the independent and joint associations of PA and DII with overweight/obesity. Moreover, we performed these analyses using a large sample representing the US adults, thereby conferring our study more statistical power to draw valid conclusion on these issues. Additionally, our study demonstrated that higher DII and more PA are still associated with risks of overweight/obesity, indicating that higher DII impacts overweight/obesity substantially. However, there are some limitations of this study. First, it is difficult to elaborate on the causal relationship between PA and DII and overweight/obesity because of the cross-sectional study. Second, the participants mainly selected from the US population may differ from those selected from other populations. Third, there may remain potential confounding factors, such as the external environmental factors, psychological factors, and genetic factors.

## 5. Conclusion

In conclusion, more leisure-time PA and walk/bicycle-time PA are associated with lower risk of overweight/obesity, and higher DII is associated with higher risk of overweight/obesity. In addition, higher DII impacts overweight/obesity substantially: once the DII score reached Q4, there is still risks of overweight/obesity even if PA is performed.

## Abbreviations

NCDs: noncommunicable diseases
PA: Physical activity
DII: dietary inflammatory index
BMI: body mass index
OR: odds ratio
CI: confidence interval

## Supplementary information

**Additional file 1:** **Supplementary Figure F1** Multivariate adjusted smoothing spline plots of PA time and BMI among female participants. This model was adjusted for age, race/ethnicity, family poverty income ratio, education, marital status, smoking, and drinking. (A) total-time PA; (B) leisure-time PA; (C) walk/bicycle-time PA; (D) work-time PA.

**Additional file 2:** **Supplementary Figure F2** Multivariate adjusted smoothing spline plots of PA time and BMI among male participants. This model was adjusted for age, race/ethnicity, family poverty income ratio, education, marital status, smoking, and drinking. (A) total-time PA; (B) leisure-time PA; (C) walk/bicycle-time PA; (D) work-time PA.

**Additional file 3:** **Supplementary Figure F3** Multivariate adjusted smoothing spline plots of DII and BMI among female participants. This model was adjusted for age, race/ethnicity, family poverty income ratio, education, marital status, smoking, and drinking.

**Additional file 4:** **Supplementary Figure F4** Multivariate adjusted smoothing spline plots of DII and BMI among male participants. This model was adjusted for age, race/ethnicity, family poverty income ratio, education, marital status, smoking, and drinking.

**Additional file 5:** **Supplementary Table S1** General characteristics of participants by sex and weight status (Mean ± SE/N(weighted%)).

**Additional file 6:** **Supplementary Table S2** Association of physical activity with overweight/obese by sex.

**Additional file 7:** **Supplementary Table S3** Association of dietary inflammatory index with overweight/obese by sex.

**Additional file 8:** **Supplementary Table S4** Joint association of physical activity and dietary inflammatory index with overweight/obese by sex.

## Declarations

### Data availability statement

This study used the NHANES publicly available data, https://wwwn.cdc.gov/nchs/nhanes.
