# Supplementary figures and images for: Association of physical activity and dietary inflammatory index with overweight/obesity in US adults: NHANES 2007–2018
Source: Environ Health Prev Med. 2023 Jun 28;28:40. doi: 10.1265/ehpm.23-00016 (PMC10331001; doi:10.1265/ehpm.23-00016)

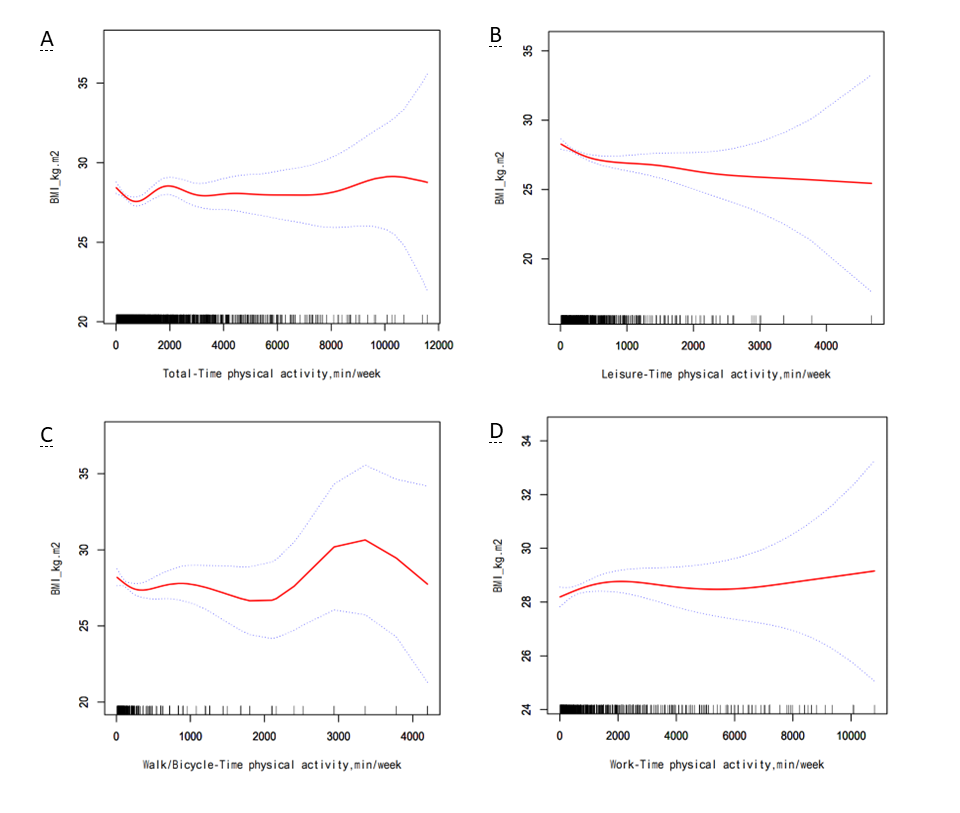

Supplement: Supplementary file 1 — Additional file 1: Supplementary Figure F1 Multivariate adjusted smoothing spline plots of PA time and BMI among female participants. This model was adjusted for age, race/ethnicity, family poverty income ratio, education, marital status, smoking, and drinking. (A) total-time PA; (B) leisure-time PA; (C) walk/bicycle-time PA; (D) work-time PA. [file ehpm-28-040-s001.tif]

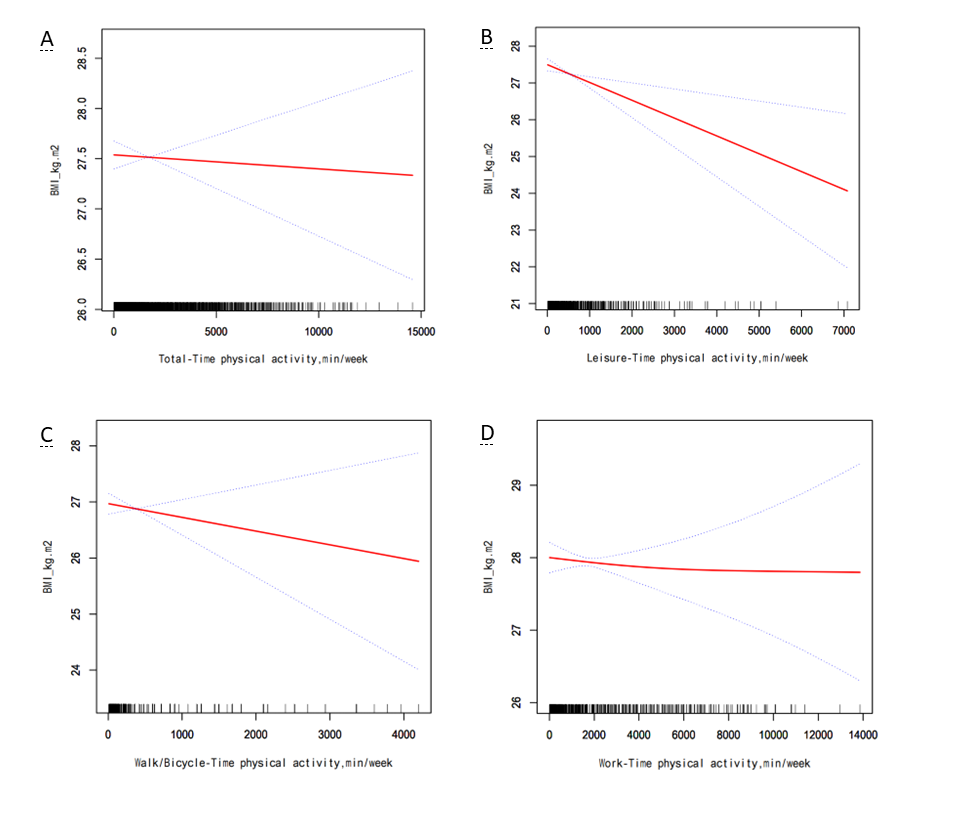

Supplement: Supplementary file 2 — Additional file 2: Supplementary Figure F2 Multivariate adjusted smoothing spline plots of PA time and BMI among male participants. This model was adjusted for age, race/ethnicity, family poverty income ratio, education, marital status, smoking, and drinking. (A) total-time PA; (B) leisure-time PA; (C) walk/bicycle-time PA; (D) work-time PA. [file ehpm-28-040-s002.tif]

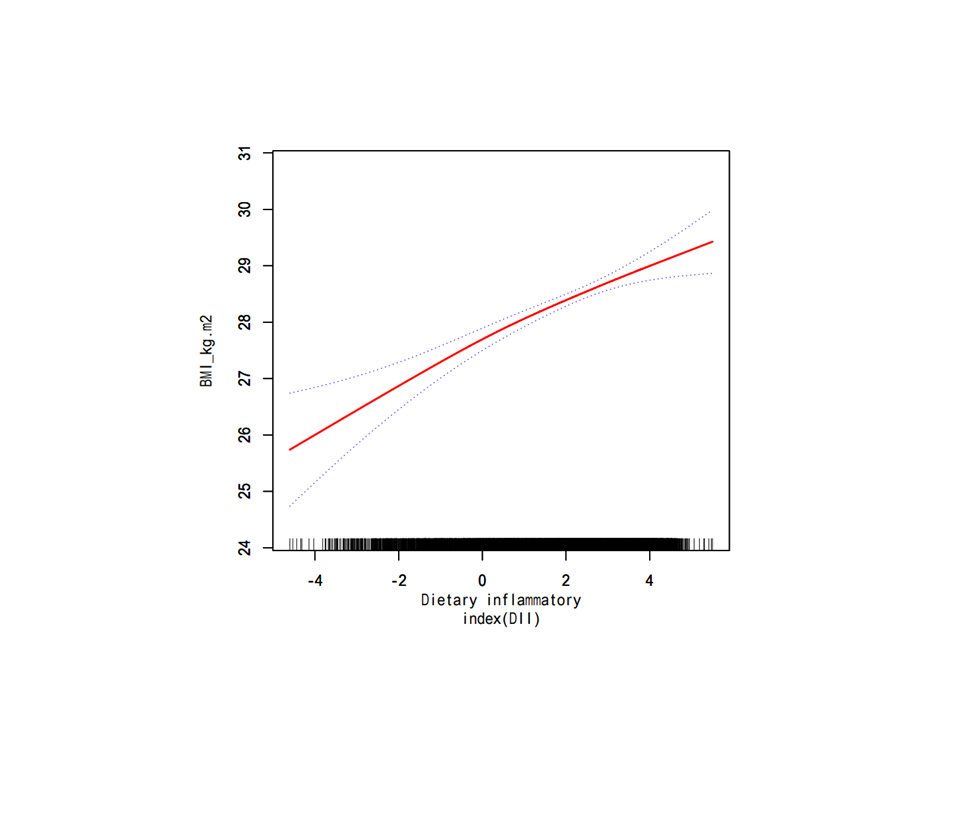

Supplement: Supplementary file 3 — Additional file 3: Supplementary Figure F3 Multivariate adjusted smoothing spline plots of DII and BMI among female participants. This model was adjusted for age, race/ethnicity, family poverty income ratio, education, marital status, smoking, and drinking. [file ehpm-28-040-s003.tif]

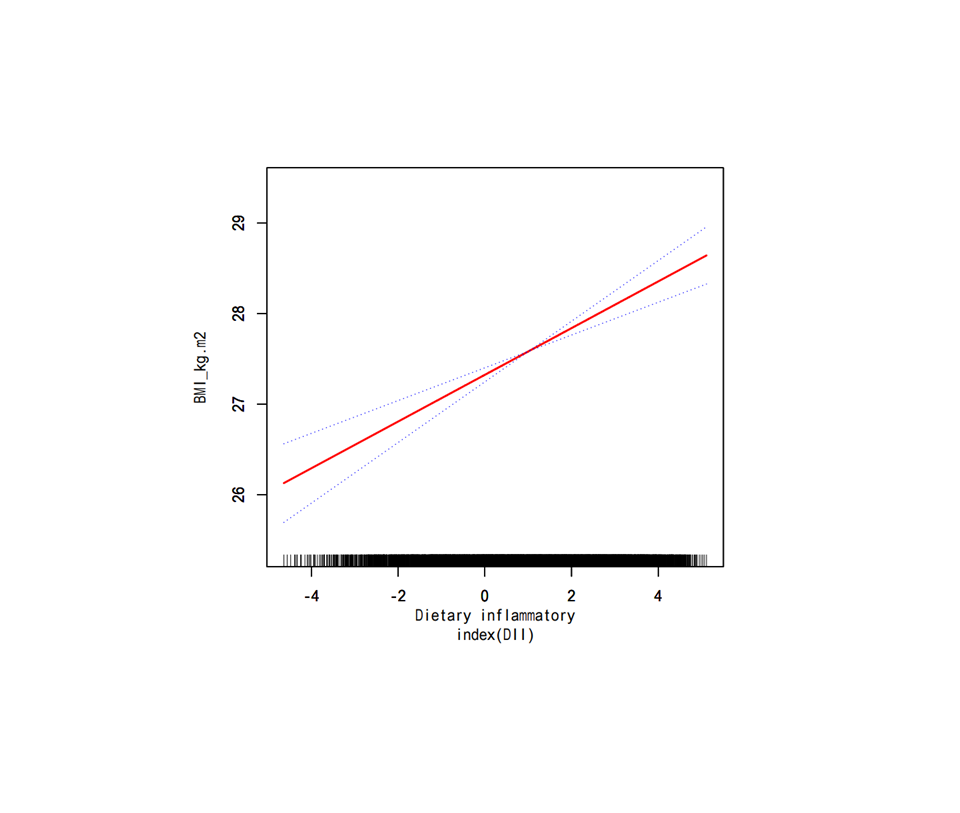

Supplement: Supplementary file 4 — Additional file 4: Supplementary Figure F4 Multivariate adjusted smoothing spline plots of DII and BMI among male participants. This model was adjusted for age, race/ethnicity, family poverty income ratio, education, marital status, smoking, and drinking. [file ehpm-28-040-s004.tif]
