# Supplementary material for: Association of physical activity and dietary inflammatory index with overweight/obesity in US adults: NHANES 2007–2018
Source: Environ Health Prev Med. 2023 Jun 28;28:40. doi: 10.1265/ehpm.23-00016 (PMC10331001; doi:10.1265/ehpm.23-00016)
Supplement: Supplementary file 5 — Additional file 5: Supplementary Table S1 General characteristics of participants by sex and weight status (Mean ± SE/N(weighted%)). [file ehpm-28-040-s005.docx]

**Supplementary Table S1 General characteristics of participants by sex and weight status (Mean ± SE/N(weighted%))**

| **Characteristics** | **Female** |  |  | **Male** |  |
| --- | --- | --- | --- | --- | --- |
|  | **Normal weight** | **Overweight/**  **obese** |  | **Normal weight** | **Overweight/**  **obese** |
| **Overall** | 2095 (58.2) | 3545 (49.7) |  | 1732 (41.8) | 3351 (50.3) |
| **Age** | 39.7 ± 15.0 (40.7) | 41.6 ± 14.5 (42.1) |  | 38.9 ± 15.7 (37.8) | 41.5 ± 14.6 (41.0) |
| **Race/ethnicity** |  |  |  |  |  |
| Hispanic group | 419 (9.9) | 1102 (16.5) |  | 292 (10.6) | 1004 (18.1) |
| Non-Hispanic White | 1041(74.8) | 1462 (66.9) |  | 760 (68.8) | 1460 (67.7) |
| Non-Hispanic Black | 235 (5.6) | 733 (11.5) |  | 340 (10.2) | 553 (8.1) |
| Other Race | 400 (9.7) | 248 (5.0) |  | 340 (10.5) | 334 (6.2) |
| **Family poverty income ratio** |  |  |  |  |  |
| <1.30 | 495 (15.7) | 1186 (22.3) |  | 571 (23.8) | 894 (17.1) |
| 1.30-3.49 | 710 (29.3) | 1311 (36.2) |  | 606 (32.2) | 1273 (34.9) |
| ≥3.50 | 890 (55.0) | 1048 (41.5) |  | 555 (44.0) | 1184 (48.0) |
| **Education** |  |  |  |  |  |
| Less than high school graduate | 217 (6.6) | 674 (12.2) |  | 327 (13.4) | 678 (13.3) |
| High school graduate or GED | 341 (15.0) | 744 (21.0) |  | 395 (22.2) | 791 (23.5) |
| Some college or above | 1537 (78.4) | 2127 (66.8) |  | 1010 (64.4) | 1882 (63.1) |
| **Marital status** |  |  |  |  |  |
| Married | 1067 (55.4) | 1673 (52.4) |  | 763 (45.2) | 1876 (59.7) |
| Never married | 556 (23.6) | 771 (19.4) |  | 606 (35.9) | 747 (21.9) |
| Widowed | 58 (2.1) | 154 (3.6) |  | 20 (0.7) | 44 (0.8) |
| Divorced | 194 (9.4) | 436 (12.4) |  | 106 (5.8) | 211 (6.0) |
| Separated | 56 (2.1) | 129 (2.4) |  | 43 (1.8) | 84 (1.7) |
| Living with partner | 164 (7.4) | 381 (9.6) |  | 192 (10.6) | 388 (9.9) |
| **Smoking** |  |  |  |  |  |
| Never | 1471 (67.7) | 2343 (64.2) |  | 917 (55.3) | 1792 (55.4) |
| Former | 303 (17.3) | 566 (18.4) |  | 293 (18.0) | 832 (25.4) |
| Now | 321 (15.0) | 636 (17.5) |  | 522 (26.8) | 727 (19.2) |
| **Drinking** |  |  |  |  |  |
| Never | 324 (10.5) | 605 (13.7) |  | 166 (8.2) | 241 (5.8) |
| Former | 163 (6.3) | 402 (10.2) |  | 184 (8.6) | 394 (10.4) |
| Mild | 676 (34.6) | 994 (30.8) |  | 698 (43.5) | 1292 (41.4) |
| Moderate | 507 (28.4) | 801 (23.9) |  | 232 (13.6) | 462 (14.1) |
| Heavy | 425 (20.2) | 743 (21.4) |  | 452 (26.2) | 962 (28.3) |
| **Total-Time PA** |  |  |  |  |  |
| Inactive | 677 (26.5) | 1428 (37.5) |  | 382 (18.9) | 788 (20.9) |
| Active | 1418 (73.5) | 2117 (62.5) |  | 1350 (81.1) | 2563 (79.1) |
| **Leisure-Time PA** |  |  |  |  |  |
| Inactive | 1154 (47.8) | 2355 (63.3) |  | 883 (48.0) | 1855 (52.1) |
| Active | 941 (52.2) | 1190 (36.7) |  | 849 (52.0) | 1496 (47.9) |
| **Walk/Bicycle-Time PA** |  |  |  |  |  |
| Inactive | 1770 (85.4) | 3099 (88.8) |  | 1334 (80.0) | 2815 (85.4) |
| Active | 325 (14.6) | 446 (11.2) |  | 398 (20.0) | 536 (14.6) |
| **Work-Time PA** |  |  |  |  |  |
| Inactive | 1460 (67.1) | 2387 (65.3) |  | 965 (52.6) | 1740 (50.8) |
| Active | 635 (32.9) | 1158 (34.7) |  | 767 (47.4) | 1611 (49.2) |
| **Dietary inflammatory**  **index (quartile)** |  |  |  |  |  |
| Q1 | 532 (27.9) | 624 (18.6) |  | 638 (40.0) | 1105 (35.0) |
| Q2 | 539 (26.0) | 816 (23.7) |  | 465 (26.6) | 939 (27.1) |
| Q3 | 515 (24.8) | 973 (27.9) |  | 356 (19.4) | 731 (21.2) |
| Q4 | 509 (21.2) | 1132 (29.8) |  | 273 (14.1) | 576 (16.8) |

DII quartile ranges: Quartile 1 = -4.634 to 0.061, Quartile 2 = 0.062-1.625, Quartile 3 = 1.626-2.948, Quartile 4 = 2.949-5.502.
