# Supplementary material for: Association of physical activity and dietary inflammatory index with overweight/obesity in US adults: NHANES 2007–2018
Source: Environ Health Prev Med. 2023 Jun 28;28:40. doi: 10.1265/ehpm.23-00016 (PMC10331001; doi:10.1265/ehpm.23-00016)
Supplement: Supplementary file 6 — Additional file 6: Supplementary Table S2 Association of physical activity with overweight/obese by sex. [file ehpm-28-040-s006.docx]

**Supplementary Table S2 Association of physical activity with overweight/obese by sex**

| **Physical activity (PA)** | **Female** |  | **Male** |  |
| --- | --- | --- | --- | --- |
|  | OR (95%CI) |  | OR (95%CI) |  |
| **Total-Time PA** |  |  |  |  |
| Inactive | 1.000 (reference) |  | 1.000 (reference) |  |
| Active | **0.677 (0.581, 0.790)** |  | 0.960 (0.810, 1.137) |  |
| **Leisure-Time PA** |  |  |  |  |
| Inactive | 1.000 (reference) |  | 1.000 (reference) |  |
| Active | **0.625 (0.542, 0.721)** |  | 0.901 (0.753, 1.078) |  |
| **Walk/Bicycle-Time PA** |  |  |  |  |
| Inactive | 1.000 (reference) |  | 1.000 (reference) |  |
| Active | **0.728 (0.584, 0.908)** |  | 0.826 (0.672, 1.015) |  |
| **Work-Time PA** |  |  |  |  |
| Inactive | 1.000 (reference) |  | 1.000 (reference) |  |
| Active | 1.092 (0.907, 1.315) |  | 1.046 (0.889, 1.232) |  |

OR: adjusted for age, race/ethnicity, family poverty income ratio, education, marital status, smoking, and drinking.
