# Supplementary material for: Association of physical activity and dietary inflammatory index with overweight/obesity in US adults: NHANES 2007–2018
Source: Environ Health Prev Med. 2023 Jun 28;28:40. doi: 10.1265/ehpm.23-00016 (PMC10331001; doi:10.1265/ehpm.23-00016)
Supplement: Supplementary file 7 — Additional file 7: Supplementary Table S3 Association of dietary inflammatory index with overweight/obese by sex. [file ehpm-28-040-s007.docx]

**Supplementary Table S3 Association of dietary inflammatory index with overweight/obese by sex**

| **Dietary inflammatory**  **index (quartile)** | **Female** |  | **Male** |  |
| --- | --- | --- | --- | --- |
|  | OR (95%CI) |  | OR (95%CI) |  |
| **Q1** | 1.000 (reference) |  | 1.000 (reference) |  |
| **Q2** | **1.319 (1.021, 1.706)** |  | 1.169 (0.932, 1.468) |  |
| **Q3** | **1.564 (1.220, 2.005)** |  | **1.370 (1.087, 1.726)** |  |
| **Q4** | **1.803 (1.435, 2.264)** |  | **1.680 (1.318, 2.141)** |  |

OR: adjusted for age, race/ethnicity, family poverty income ratio, education, marital status, smoking, and drinking.

DII quartile ranges: Quartile 1 = -4.634 to 0.061, Quartile 2 = 0.062-1.625, Quartile 3 = 1.626-2.948, Quartile 4 = 2.949-5.502.
