# Supplementary material for: Association of physical activity and dietary inflammatory index with overweight/obesity in US adults: NHANES 2007–2018
Source: Environ Health Prev Med. 2023 Jun 28;28:40. doi: 10.1265/ehpm.23-00016 (PMC10331001; doi:10.1265/ehpm.23-00016)
Supplement: Supplementary file 8 — Additional file 8: Supplementary Table S4 Joint association of physical activity and dietary inflammatory index with overweight/obese by sex. [file ehpm-28-040-s008.docx]

**Supplementary Table S4 Joint association of physical activity and dietary inflammatory index with overweight/obese by sex**

| **Physical activity (PA)** | **DII**  **(quartile)** | **Female** |  | **Male** |  |
| --- | --- | --- | --- | --- | --- |
|  |  | OR (95%CI) |  | OR (95%CI) |  |
| **Total-Time PA** |  |  |  |  |  |
| Inactive | Q1 | 1.000 (reference) |  | 1.000 (reference) |  |
|  | Q2 | 1.193 (0.803, 1.774) |  | 1.010 (0.715, 1.424) |  |
|  | Q3 | 1.312 (0.877, 1.964) |  | 1.062 (0.742, 1.518) |  |
|  | Q4 | **1.380 (0.933, 2.040)** |  | 1.162 (0.791, 1.708) |  |
| Active | Q1 | 1.000 (reference) |  | 1.000 (reference) |  |
|  | Q2 | **1.389 (1.042, 1.851)** |  | **1.226 (1.031, 1.459)** |  |
|  | Q3 | **1.725 (1.294, 2.299)** |  | **1.398 (1.152, 1.696)** |  |
|  | Q4 | **2.087 (1.561, 2.792)** |  | **1.542 (1.244, 1.911)** |  |
| **Leisure-Time PA** |  |  |  |  |  |
| Inactive | Q1 | 1.000 (reference) |  | 1.000 (reference) |  |
|  | Q2 | 1.279 (0.946, 1.728) |  | 1.175 (0.943, 1.464) |  |
|  | Q3 | **1.484 (1.116, 1.973)** |  | **1.322 (1.047, 1.671)** |  |
|  | Q4 | **1.525 (1.180, 1.972)** |  | **1.501 (1.169, 1.927)** |  |
| Active | Q1 | 1.000 (reference) |  | 1.000 (reference) |  |
|  | Q2 | 1.377 (0.962, 1.971) |  | 1.170 (0.941, 1.455) |  |
|  | Q3 | **1.715 (1.182, 2.489)** |  | **1.294 (1.009, 1.660)** |  |
|  | Q4 | **2.364 (1.608, 3.476)** |  | **1.336 (1.003, 1.778)** |  |
| **Walk/Bicycle-Time PA** | |  |  |  |  |
| Inactive | Q1 | 1.000 (reference) |  | 1.000 (reference) |  |
|  | Q2 | **1.371 (1.053, 1.786)** |  | 1.107 (0.933, 1.316) |  |
|  | Q3 | **1.613 (1.218, 2.136)** |  | 1.200 (0.993, 1.449) |  |
|  | Q4 | **1.840 (1.421, 2.382)** |  | **1.314 (1.068, 1.619)** |  |
| Active | Q1 | 1.000 (reference) |  | 1.000 (reference) |  |
|  | Q2 | 1.027 (0.595, 1.774) |  | **1.469 (1.025, 2.104)** |  |
|  | Q3 | 1.150 (0.643, 2.058) |  | **1.844 (1.254, 2.711)** |  |
|  | Q4 | 1.598 (0.956, 2.670) |  | **2.165 (1.422, 3.295)** |  |
| **Work-Time PA** |  |  |  |  |  |
| Inactive | Q1 | 1.000 (reference) |  | 1.000 (reference) |  |
|  | Q2 | 1.297 (0.976, 1.722) |  | **1.284 (1.042, 1.583)** |  |
|  | Q3 | **1.554 (1.159, 2.084)** |  | **1.289 (1.024, 1.623)** |  |
|  | Q4 | **1.683 (1.295, 2.188)** |  | **1.413 (1.094, 1.824)** |  |
| Active | Q1 | 1.000 (reference) |  | 1.000 (reference) |  |
|  | Q2 | 1.344 (0.917, 1.971) |  | 1.042 (0.828, 1.311) |  |
|  | Q3 | **1.592 (1.080, 2.346)** |  | **1.329 (1.034, 1.707)** |  |
|  | Q4 | **2.018 (1.290, 3.158)** |  | **1.468 (1.117, 1.928)** |  |

OR: adjusted for age, race/ethnicity, family poverty income ratio, education, marital status, smoking, and drinking.

DII quartile ranges: Quartile 1 = -4.634 to 0.061, Quartile 2 = 0.062-1.625, Quartile 3 = 1.626-2.948, Quartile 4 = 2.949-5.502.
